# Evaluation of the Performance of Various Diagnostic Modalities Available for the Detection of Scrub Typhus in Acute Undifferentiated Febrile Illness (AUFI) Cases at a Teaching Hospital in North Chhattisgarh, India

**DOI:** 10.7759/cureus.78977

**Published:** 2025-02-14

**Authors:** Pratiksha P Dwivedi, Arvind K Singh, Ramanesh Murthy, Sourabh Dwivedi, Akash R Verma

**Affiliations:** 1 Department of Microbiology, Rajmata Shrimati Devendra Kumari Singhdeo Government Medical College, Ambikapur, Ambikapur, IND; 2 Department of Microbiology, Chhattisgarh Institute of Medical Sciences, Bilaspur, Bilaspur, IND; 3 Department of Biochemistry, Shyam Shah Medical College, Rewa, Rewa, IND; 4 Department of Community Medicine, Rajmata Shrimati Devendra Kumari Singhdeo Government Medical College, Ambikapur, Ambikapur, IND

**Keywords:** aufi, elisa, rapid diagnostic test (rdt), rt-pcr, scrub typhus, zoonosis

## Abstract

Introduction: Due to the lack of adequate data on the effectiveness of diagnostic methods and the ambiguous clinical symptoms that overlap with other febrile illnesses, diagnosing scrub typhus is difficult. This study aims to compare the accuracy of various investigations required for the diagnosis of scrub typhus like immunoglobulin G/immunoglobulin M (IgG/IgM) rapid test, IgM enzyme-linked immunosorbent assay (ELISA), and real-time polymerase chain reaction (RT-PCR) from a patient’s serum.

Methods: This is a prospective study that includes all clinically suspected patients who visited the Outpatient Department (OPD) of Medicine and were admitted to the Medicine wards and Intensive Care Units of Rajmata Shrimati Devendra Kumari Singhdeo Government Medical College, Ambikapur, Chhattisgarh, India. The patients’ samples were tested initially using the IgG/IgM rapid test, further confirmed by ELISA, and then subjected to RT-PCR for final confirmation.

Results: A total of 1,620 cases of acute undifferentiated febrile illness were tested, of which 82 tested positive for scrub typhus IgM rapid test. These 82 cases were further tested for confirmation using IgM ELISA, which showed 110 positive results. Additionally, RT-PCR was applied to all 1,620 samples using the DIAGsure Tropical Fever Panel Kit (3B BlackBio Dx Limited, Bhopal, India), resulting in 98 samples testing positive for scrub typhus. Both the ELISA and the rapid diagnostic test offer high capacity for discrimination, with sensitivity and specificity of 92.40%, 93.18%, and 99.20%, 98.17%, respectively (10.9% of cases came positive in serology which was negative in RT-PCR). It can be due to its nonspecific binding with antibodies of other febrile illnesses such as malaria, enteric fever, pulmonary tuberculosis, leptospirosis, etc.

Conclusion: RT-PCR has shown excellent results with a sensitivity of >95% and specificity of >99%. Given its high sensitivity and specificity, along with clinical findings, RT-PCR is highly effective in detecting scrub typhus, especially for diagnosing early stages of the disease in cases of acute febrile illness with a duration of less than seven days. In reference labs, RT-PCR is the primary method for confirmation. This paper offers a thorough assessment of all the diagnostic tests for scrub typhus that are now accessible in a setting with limited resources, such as our north Chhattisgarh region.

## Introduction

The most common febrile illness caused due to rickettsial infection in the Indian subcontinent is scrub typhus [1]. This zoonotic disease is brought on by the intracytosolic bacteria *Orientia tsutsugamushi.* Humans are the contingent host of species of *Leptotrombidium* and stalemate host of *O. tsutsugamushi* and it gets passed down to humans following the bite of *Leptotrombidium deliense*, which is Chigger mite species and main vector of scrub typhus. Rodents are used as hosts by the mites [2] and therefore higher incidence is noted in rodent carriers [3]. In the Tsutsugamushi Triangle (which extends from Northern Japan and far Eastern Russia in the North to Northern Australia in the South and to Pakistan in the west), it is one of the most prevalent rickettsial illnesses and a reemerging infectious disease [1]. It is endemic in Southeast Asia. The higher prevalence is noted in rural areas of Southeast Asia including Korea, Thailand, India, Russia, Australia, Japan, and the Pacific Islands [1]. The burden of this disease in India is still in dilemma, but largely we have failed to spot this as a trouble. In India, by the time of the Second World War, i.e., after early epidemics in the states of Bengal and Assam, re-emergence of cases occurred there in recent years because wood for cooking food was commonly stored in backyards near kitchens which are often outdoors, which led to increase in population of rodents over there and rodents influence mite population density and serve as a reservoir for the agent [4] and from other states in India such as Jammu and Kashmir, Himachal Pradesh, Tamil Nadu, Uttaranchal, Rajasthan, Bihar, Kerela, Maharashtra, Meghalaya, Karnataka, and West Bengal [5,6]. The most challenging part is its diagnosis because of varied clinical manifestations and hence it becomes difficult to discriminate scrub typhus from other febrile illnesses of undifferentiated type like malaria, dengue, urinary tract infection, leptospirosis, typhoid, etc. Additionally, the low index of suspicion and the limited availability of diagnostic resources further complicate timely and accurate identification [7].

High-grade fever, headache, myalgia, lymphadenopathy, eschar, rash, and other symptoms are some of the earliest signs of an acute undifferentiated febrile illness (AUFI) [8]. Acute respiratory distress syndrome (ARDS), acute kidney injury (AKI), myocarditis, pneumonia, gastrointestinal hemorrhage, meningoencephalitis, hepatitis, and other more serious illnesses can develop from scrub typhus. However, the pathognomic manifestation of scrub typhus is the presence of eschar at the bite site of the mite. Approximately, 33% of patients suffer from multi-organ dysfunction syndrome (MODS) during the entire disease course and potentially fatal outcome [5]. Another important clinical manifestation to be considered is thrombocytopenia [9,10] which is one of the causes of MODS [11,12].

Deficient statistical analysis and proper health records in outbreak regions signify a gap in attainments in this spurned illness. Timely diagnosis of scrub typhus is crucial for effective patient management and guiding appropriate therapy, thereby preventing potential complications. In India, on a wider scale, scrub typhus is diagnosed serologically by non-specific Weil Felix agglutination test or by enzyme-linked immunosorbent assay (ELISA), or in some centers by a polymerase chain reaction (PCR) and immunofluorescent assay [13,14]. The most preferred sample for PCR analysis is blood (buffy coat or whole blood). Although eschar is a definitive sign of scrub typhus, it is considered a less preferred sample for PCR analysis [15,16].

The accuracy, sensitivity, and specificity of the existing laboratory tests for verifying the diagnosis of scrub typhus must be examined because there are no practical, accurate, sensitive, and reasonably priced diagnostic modalities in areas where the disease is endemic.

## Materials and methods

This 21-month prospective study was conducted at Rajmata Shrimati Devendra Kumari Singhdeo Government Medical College (RSDKS GMC), Ambikapur, Ambikapur, North Chhattisgarh, India between January 2023 and September 2024.

Inclusion criteria

This study includes all clinically suspected patients who have visited Outpatient Departments (OPDs) of various departments mainly medicine and were admitted to medicine wards and Intensive Care Units of the district hospital in Ambikapur attached with RSDKS GMC among all age groups, with AUFI of fever lasting fewer than 14 days duration with no localizing signs and symptoms for any other illness, before the initiation of doxycycline therapy and who had signed written detailed and informed consent.

Exclusion criteria

Exclusion was determined through a detailed history, comprehensive clinical evaluation, and appropriate laboratory investigations, including complete blood count, three consecutive blood smears for malarial parasites, blood, urine, and stool cultures for enteric fever, and sputum/endotracheal cultures to rule out viral pharyngitis or respiratory tract infections. Additionally, serology for dengue, enteric fever, and leptospirosis, acid-fast bacilli smear using the Ziehl-Neelsen method, chest X-ray, abdominal ultrasound (USG), and contrast-enhanced computed tomography (CECT) of the thorax and abdomen were performed when required. Patients with a confirmed diagnosis of malaria, urinary tract infection, dengue, enteric fever, leptospirosis, or viral fever were excluded.

Sample collection and testing

Serum and whole blood samples were collected in red-capped plastic vacutainers containing a clot activator, without any anticoagulants, preservatives, or separator material. All 1620 samples were subjected to all the tests available at our center and the results obtained are compared to calculate the relative sensitivities and specificities. The tests performed included a lateral flow immunochromatographic assay using the Progen^TM^ (Progen, Germany) and Trust Line IgG/IgM Rapid Test Kit (Athenese-Dx, Chennai, India), as well as the InBios IgM (InBios International, Inc., Seattle, Washington, USA) and Trustwell IgM ELISA Kit (Athenese-Dx). Since the study was conducted in a government setup, the use of kits from different manufacturers depended on availability. Additionally, DIAGsure Tropical Fever Panel Real-Time PCR Kit (Biotron Healthcare, Mumbai, India) (which detects dengue, chikungunya, typhoid, malaria, scrub typhus, and leptospirosis) was used, with all tests performed according to the manufacturers’ instructions. Ethical approval for this study was granted by the Institutional Ethical Committee of RSDKS GMC, Ambikapur (No. IEC/13/GMC Ambikapur). Biochemical parameters including fasting blood sugar, liver function test, renal function test, and electrolytes were done and recorded.

Statistical analysis

Statistics were calculated using the Statistical Package for the Social Sciences (IBM SPSS Statistics for Windows, IBM Corp., Version 20.0, Armonk, NY).

## Results

In this prospective investigation, all 1620 individuals who presented with a febrile illness similar to typhus were tested using all three diagnostic modalities accessible in our setup, and the results obtained were compared to calculate the relative sensitivities and specificities.

**Figure 1:**
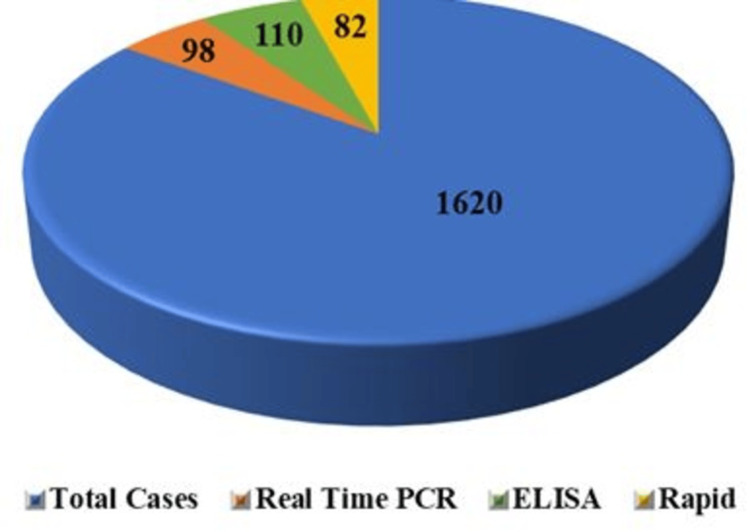
Positive Cases Identified Through Various Diagnostic Modalities PCR: polymerase chain reaction; ELISA: enzyme-linked immunosorbent assay

**Figure 2:**
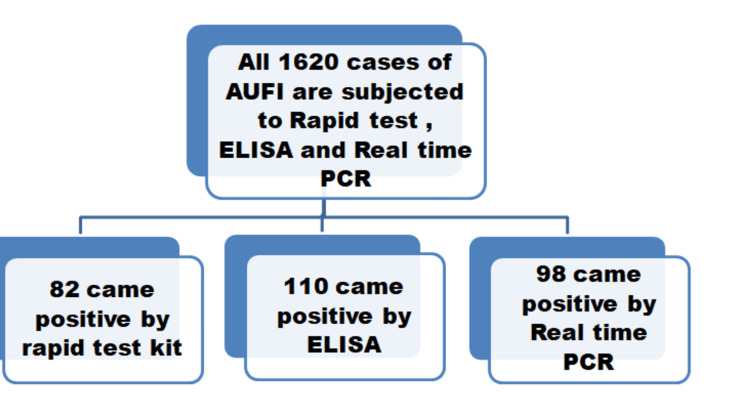
Flow Diagram of the Study AUFI: acute undifferentiated febrile illness; ELISA: enzyme-linked immunosorbent assay; PCR: polymerase chain reaction

Initially, all 1620 samples were subjected to IgG/IgM rapid tests (lateral chromatographic immunoassay) using the Progen^TM^ IgG/IgM Rapid test kit and Trust Line IgG/IgM Rapid test kit, resulting in 82 positive cases. Subsequently, all 1620 samples underwent ELISA testing for confirmation using the Trustwell IgM ELISA test kit and InBios IgM ELISA test kit, yielding 110 positive results. For further analysis, real-time PCR was conducted on all 1620 samples using the DIAGsure Tropical Fever Panel Kit, which identified 98 positive cases of scrub typhus (Figures 1-2).

To compare the diagnostic accuracy, we have compared the sensitivity, specificity, positive predictive value, and negative predictive value of the rapid test and ELISA with the results of real-time PCR, and also calculated Pearson's chi-squared test and p-value to test the significance of our study using the SPSS software (Table 1).

**Table 1:** Diagnostic Precision of Assays Used for Scrub Typhus Diagnosis in Our Setup ELISA: enzyme-linked immunosorbent assay

	Sensitivity	Specificity	Positive Predictive Value	Negative Predictive Value	Pearson's Chi-Square Test	P-value
ELISA	92.40%	99.20%	89.09%	99.40%	1243.048	<0.001
Rapid test	93.18%	98.17%	74.54%	99.60%	1315.04	<0.001

**Figure 3:**
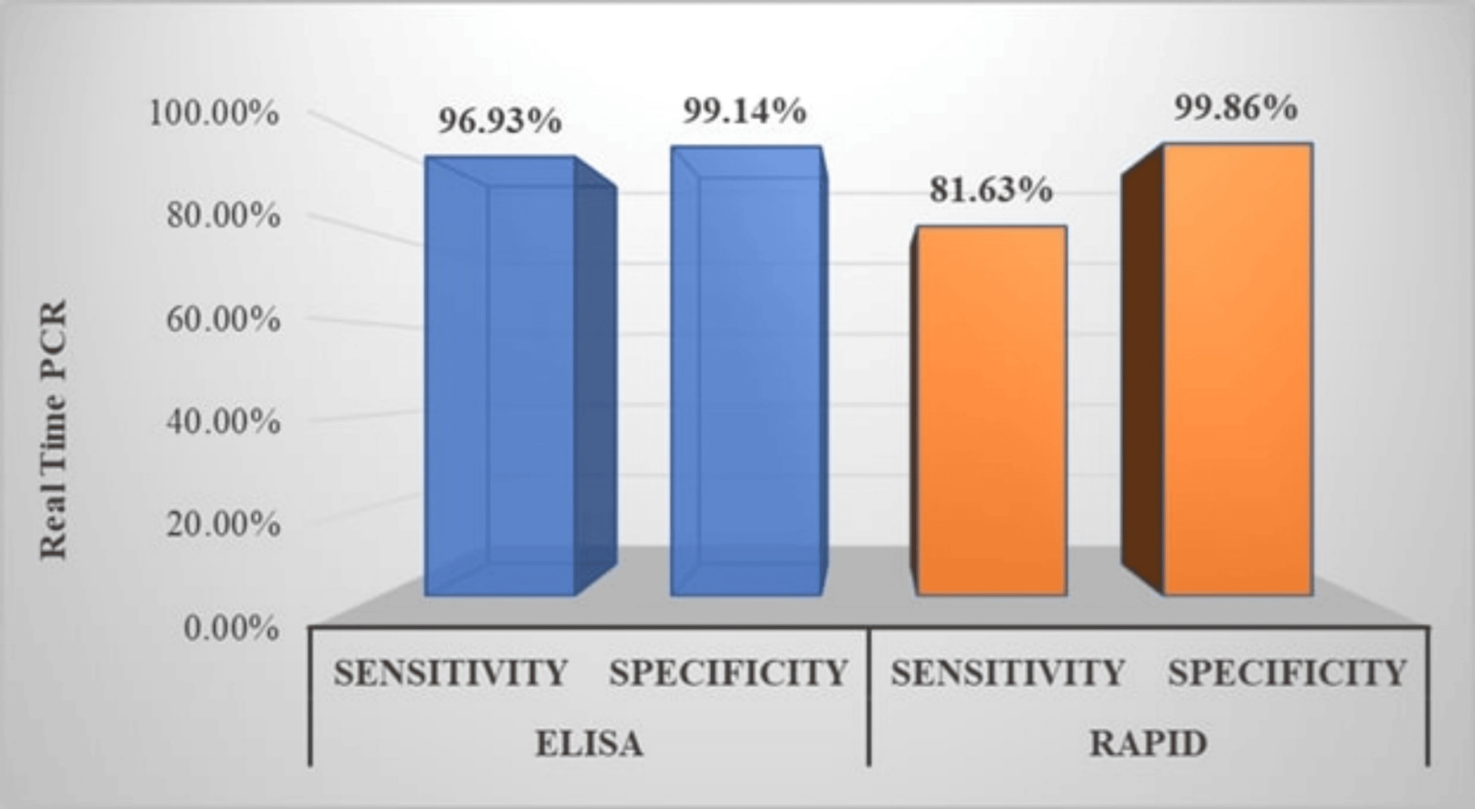
Relative Sensitivity and Specificity of Rapid Test and ELISA in Comparison With Real-Time PCR PCR: polymerase chain reaction; ELISA: enzyme-linked immunosorbent assay

**Figure 4:**
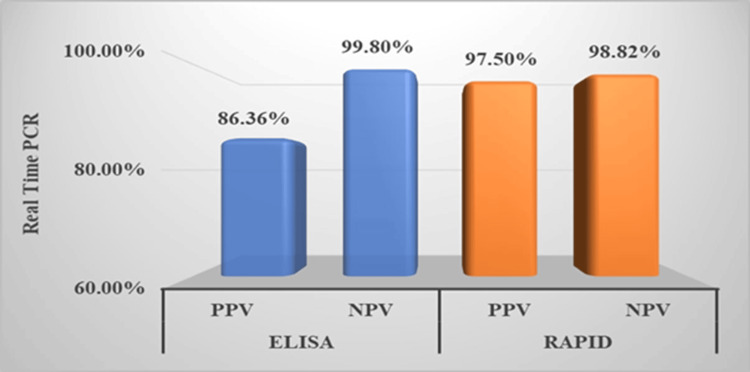
Relative Positive Predictive Value (PPV) and Negative Predictive Value (NPV) of Rapid Test and ELISA in Comparison With Real-Time PCR PCR: polymerase chain reaction; ELISA: enzyme-linked immunosorbent assay

The ELISA test was found to have a sensitivity of 92.40% (n=98) and a specificity of 99.20% (n=1502). The positive predictive value was 89.09%, and the negative predictive value was 99.40%. Meanwhile, the rapid test showed a sensitivity of 93.18% (n=82) and specificity of 98.17% (n=1516), with a positive predictive value of 74.54% and a negative predictive value of 99.60%. The cumulative false positivity rate for both the IgM ELISA kits used for scrub typhus was 10.9%. The analytical sensitivity of the DIAGsure Tropical Fever Panel Kit for real-time PCR is 20 nucleic acid copies per reaction. The clinical sensitivity is >95%, and the specificity is >99%, as reported by the manufacturing company. A p-value of <0.001 for both the ELISA and real-time PCR tests indicates a statistically significant difference in the observed results (Figures 3-4).

## Discussion

The most common rickettsial infection across the world is scrub typhus which bears the potency to cause fatality, affecting around one million people per year across the world [17], and it becomes formidable to diagnose scrub typhus infection because of the broader clinical picture conjoining with other undifferentiated febrile illness, poor cognizance and paucity of adequate data on execution of diagnostic modalities [7]. The mainstay of diagnosis still lies in serological testing because of its convenient performance. The standard serological test for the detection of antibodies of *O. tsutsugamushi* is an immunofluorescent assay [18].

The performance of three diagnostic modalities that were accessible at our center for the identification of scrub typhus cases in their acute phase of febrile illness, or duration of less than 14 days, both on an outpatient basis and after patient admission, has been comprehensively assessed in this study. In this prospective study of 21 months duration conducted in RSDKS GMC, Ambikapur located in the north Chhattisgarh region where scrub typhus is endemic, we interpreted that both the ELISA and the rapid diagnostic test have excellent discernative potential with sensitivities and specificities of 92.40%, 93.18%, and 99.20%, 98.17%, respectively. We also found that each assay was sensitive enough to detect anti-*O. tsutsugamushi* IgM and total antibodies for diagnosing the acute phase of scrub typhus infection. There was an 83.67% agreement between the rapid test and real-time PCR and an 89.09% agreement between ELISA and real-time PCR. With a sensitivity of over 95% and a specificity of over 99%, real-time PCR has demonstrated promising results.

The rapid test kits used were Progen^TM^ IgG/IgM Rapid Test Kit and Trust Line IgG/IgM Rapid Test Kit, of which 82 cases came back positive. Because of its low specificity and sensitivity, Progen^TM^, the antigen suspension used in the Weil-Felix test, contained ready-to-use standardized, killed, stained, smooth, and specific antigen suspensions of Proteus OXK/OX19/OX2. These suspensions were based on antibodies that cross-reacted with the antigens of Proteus species. In their investigation, Mahajan SK et al. determined that the Weil-Felix agglutination test is specific but not very sensitive [19]. Similar results of low sensitivity and high specificity of the Weil-Felix test were observed in our investigation as well. Trust Line IgG/IgM Rapid test kit was based upon lateral flow immunochromatographic assay and has shown similar sensitivity and specificity as provided in the manufacturer’s guide.

We used an optimal optical density (OD) cutoff of 0.5 for IgM to determine ELISA positivity, as per the manufacturer’s instructions for the InBios kit. For the Trustwell ELISA kit, the cutoff was calculated according to the user’s guide provided by the company. Using a limit of 0.5 OD, InBios IgM ELISA was utilized in Thailand to assess the diagnosis of scrub typhus in admitted patients, yielding 91% specificity and 93% sensitivity [20]. A 10.9% cumulative false positivity rate was observed when using both the IgM ELISA kits for scrub typhus. The IgM antibody shows cross-reactivity with many other febrile illnesses of acute duration, such as malaria, leptospirosis, enteric fever, and pulmonary tuberculosis [21,22]. The probable cause of false positivity can be attributed to the pentavalent structure of the IgM antibody, which results in nonspecific IgM antibody binding [23]. Our study revealed that there is a type 1 error (occurs in a study in research when we reject the null hypothesis and erroneously state that the study found significant differences when there was indeed no difference) in ELISA and lower detection rate in rapid diagnostic kits. The accuracy of data points supplied by identical manufacturers varied among research, according to a 2018 meta-analysis by Saraswati K et al. [24]. However, real-time PCR has shown accurate results in detection as well as specificity and sensitivity which is in conjunction with the previous research by Kannan K et al., published in 2020, indicated that, given its complete sensitivity and specificity, it might be used to diagnose early disease with a period of less than seven days. In reference labs, real-time PCR is the primary method for confirmation [23].

This study bears some limitations that it was conducted in a resource-limited setting where a gold standard test (immunofluorescent assay) was not available for the detection of scrub typhus and kits used for rapid diagnostic tests and ELISA were taken from two different manufacturing companies.

## Conclusions

This study concludes that the ELISA test for *O. tsutsugamushi *IgM antibodies has high sensitivity and specificity but a notable type 1 error rate. The rapid detection test shows satisfactory sensitivity and specificity, with 83.67% agreement with real-time PCR, making it useful for diagnosing scrub typhus in resource-limited settings.

Real-time PCR demonstrates excellent sensitivity and specificity, making it the confirmatory method for diagnosing scrub typhus in acute febrile illness under seven days, particularly in well-equipped labs. The study evaluates various diagnostic tests crucial for improving patient outcomes in areas with limited healthcare access like north Chhattisgarh.
